# Supplementary material for: Environmental temperature is a strong driver of subspecies competition in the Drosophila microbiome
Source: eLife. 2026 Sep 18;15:RP110808. doi: 10.7554/eLife.110808 (PMC13588619; doi:10.7554/eLife.110808)
Supplement: Peer review history

# Editor report: eLife Assessment

**DOI:** 10.7554/eLife.110808.3.sa0

**Authors:** María Mercedes Zambrano

**Affiliations:** CorpoGen Colombia

**Keywords:** Solid, Valuable

This **valuable** study explores changes in the Drosophila microbiome in response to environmental temperature over more than ten years. The evidence that temperature leads to diversification of bacterial clades is **solid**, despite the need for greater clarity in defining and tracking strain competition. The work will interest researchers working with microbiomes, microbial ecology, and evolutionary biology.

# Referee report: Reviewer #1 (Public review)

**DOI:** 10.7554/eLife.110808.3.sa1

Summary:

The factors that create and maintain diversity in host-associated microbiomes remain poorly understood. A better understanding of these factors will help in the efforts to leverage the adaptive potential of the microbiome to help solve pressing problems in health and agriculture.

Experimental evolution provides a promising path forward as we can track the causes and consequences in the emergence of novel variants, but experimental evolution remains underutilized in host-microbiome interactions. Here, Gracia-Alvira utilizes a long-term experimental evolution study in Drosophila simulans under hot and cold temperature regimes to identify strain-level variation in an important fly bacterium, Lactiplantibacillus plantarum. They identify three strains of L. plantarum, which are most prevalent in their respective three temperature regimes, suggesting that these are locally adapted bacteria. Then, using a combination of genomics, in vitro, and in vivo, Gracia-Alvira et al attempt to understand the factors that led to the differentiation of the hot and cold L. plantarum and their impacts on the fly host.

Strengths:

This is an excellent use of experimental evolution to track the emergence of novelty in the microbiome. The genomic analyses are all solid and appropriate for the data sets. It is especially striking that the comparisons with the other, independent experimental evolution studies in different labs (and across continents between Portugal and South Africa) show a consistent response to temperature. Many have disregarded the microbiome as it is something that is too sensitive to seemingly innocuous variables (particularly in the fly microbiome), such that we cannot find generalities. However, this finding highlights the potential for experimental evolution to uncover these dynamics. The question of how strains emerge and are maintained is timely and is one of the key open questions in host-microbiome evolution currently.

Comments on revised version:

I thank the authors for their thoughtful responses to my concerns, and I appreciate the additional experiments to help resolve the questions about subspecies competition. The manuscript remains strongest in the genomic assessment of changes in the L. plantarum genomes, and it is striking and noteworthy that the isolates across multiple countries but same temperature conditions group together phylogenetically.

I appreciate the additional clarity also incorporated in this revision, but there are still a few key concerns that are unresolved about the microbial ecology described here. I will also note that I apologize if I missed something in the text as no line numbers were provided to point me to where the changes were incorporated in the revised manuscript.

(1) Competition has many different meanings and many different measurements (see Hart 2018 https://doi.org/10.1111/1365-2745.12954) -and incorporating the effects of competition in shaping an ecological community is, has been, and will continue to drive much research in community ecology. Measuring strain level competition is one of the major questions in host-associated microbiomes, and it is difficult-though there have been significant advances in doing so (see isogenic barcodes, e.g., Daniel 2024 doi: https://doi.org/10.1038/s41564-024-01634-9b, Ordon 2024 https://doi.org/10.1038/s41564-024-01619-8, as well as my previous suggestion to track the outcomes of competition). The inability to directly track and measure competition of the isolates remains a limitation of this manuscript. The authors' explanation of measuring competition is unusual, simplistic, and at times inconsistent.

They need to be crystal clear about their definitions, logic for making these inferences, and weaknesses in their approach. I think what the authors mean is that competition between the unevolved and C or H in their respective regimes leads to the decrease of the U clade over experimental evolution. But it is not clear how the authors are thinking about competition between C and H clades in the different temperatures.

The authors state that competition is inferred because changes in relative abundance across the time series-and this is unusual because there are alternative explanations that require no ecological interactions among sub-strains, as I described in my comments on the prior version. This is then combined with in vitro work that shows that the H and C clades can both grow in their mismatched temperature regimes-and thus I think it is to be inferred that because they can grow alone in vitro (and C isolates show lower growth than H isolates in hot temperature), then changes in the relative abundance over fly generations can be attributed to competitive interactions among C and H clades. But then the logic is inconsistent because then the authors just say that in vitro growth curves don't support the differences in relative abundance observed in the flies (lines 224-225). Then the authors argue is it about a combination of diet/sugar metabolism and temperature (line 373), which doesn't make any sense because temperature previously didn't matter (lines 224-225).

All of this is to say is that the authors need to make clear their logic to the readers-and explain these inconsistencies appropriately. To me, it suggests that there are clear methodological weaknesses that inhibit the ability to track competitive microbial dynamics. Because you can't really assess the microbial dynamics in vivo, it remains further unresolved why clade C isolates have such strong negative fitness effects on the fly but reach such high relative abundances in the C evolving flies. I find that this series of logical inconsistencies (and see my point #2) distracts from the important finding that the C and H clades evolved to utilize sugars differently from the U clade, which is an interesting finding!

(2) There are also inconsistencies in the patterns observed between the text and the figures. Some of this arises because the authors are not clear what comparisons they are making. For example, line 450 says that clade C outcompeted the other clades, which I presume means only in the cold temperature. Line 456 says that C and H isolates grow faster in the sugar-rich lab diet, but that is not really true because U and C have similar growth rates in Fig. 5, and U and H have similar growth rates in Fig. S4. The text about microbial load is a bit misleading (lines 271-273), as it is confusing that clade C is significantly higher load in both hot and cold temperatures (Fig. S6), which is counterintuitive given Fig. 4, 5, S4. But it is also overly speculative to say that these results suggest that fitness effects depend on microbial load of clade C without connecting the load to the fly fitness measures (and also given the inconsistency with the time series data from evolving lines). Please take care to more carefully phrase these statements to ensure the inference is supported by the experiment design (e.g., clarifying comparison) and statistics (e.g., ensuring agreement with what the figure shows).

(3) I understand the concern about focusing the reader on the L. plantarum strains. However, it should be clear to the readers that you did not examine the other parts of the microbiome, and that L. plantarum is often very rare in lab and wild fly populations. The data presented on Table S4 (cited line 552, I think citation at line 176 is incorrect) is confusing. If these were colonies picked and then identified, this should be explicit. If it is based off on colonies, then please clarify if this was sampled randomly or occurred when trying to enrich/focus on L. plantarum isolates. If the data was computational (e.g., Kraken to classify), then only taxa richness is not necessarily relevant, but please also include to the relative abundance of each taxa.

To me, this is relevant information to contextualize these results, particularly because you test this in both D. mel and *D. simulans* (apologies for the confusion over Mazzucco & Schlotterer 2021), and we have insight into how combinations of Lactobacillus and other taxa impact fitness (Gould PNAS 2018). If the results from *D. melanogaster* are not applicable to *D. simulans*, then the authors need to explain this. I understand if incorporating analysis of the broader microbiome is beyond the scope of this manuscript, but at least acknowledging the general rarity in Lactobacillus frequency in Drosophila microbiome and variation in fitness effects will more accurately contextualization these results.

One small point is that line 452 the citations are OK, but there are fly-specific examples to support this statement, like Gould PNAS 2018, Henry Proceedings B 2025.

# Referee report: Reviewer #2 (Public review)

**DOI:** 10.7554/eLife.110808.3.sa2

Summary:

In this manuscript, Gracia-Alvira et al. investigated how environmental temperature affects competition among members of the microbiome, with a focus on intraspecific diversity, using the Drosophila model.

Notably, the authors identified three clades of Lactiplantibacillus plantarum from a natural population of Drosophila simulans collected in Florida. They tracked the dynamics of these three bacterial clades under two temperature conditions over the course of more than ten years. Using comparative genomics and phylogeny, they showed that these three bacterial clades likely adapted to their host independently in a temperature-specific manner. Further, by combining in vitro culture and in vivo mono-association assays, they demonstrated the functional divergence of these three bacterial clades phenotypically, including their growth dynamics and effects on host fitness. Lastly, they performed pathway analysis and speculated on key genomic variance supporting such functional divergence.

Strengths:

The laboratory evolutionary experiment in response to cold or hot environmental temperature is impressive, given its more than ten years of experimental time period. This collection of achieved microbiome samples paired with the fly host data can be a valuable resource for the field.

Comments on revised version:

The revised version has addressed my major points raised in the original review.

# Referee report: Reviewer #3 (Public review)

**DOI:** 10.7554/eLife.110808.3.sa3

Summary:

The study presents an analysis of 297 pangenomes derived from 20 populations of Drosophila simulans, at 19 time points for fast-reproducing individuals in a hot environment, or at 10 time points for slow-reproducing individuals in a cold environment, over a period of more than 10 years. The authors select a particular microbial component of the pangenomes and study the dynamics of Lactiplantibacillus plantarum strains in two environments. They discover that the revealed operational taxonomic units could be divided into three phylogenetic clades, which have their own genomic and genetic features, different adaptive capabilities that depend on the environment, and have a distinct impact on the fitness of the host.

Strengths:

The authors prove that bacterial microbiome components are sensitive to the environment and could rapidly (years) be fixed in eukaryotic populations. This study establishes a tractable model that potentially enables the study of variability of the physiological influence of distinct strains of an important commensal species, Lactiplantibacillus plantarum, on the Drosophila host. It is clearly shown that this single species consists of several phylogenetically and functionally diverse strains. The authors did not limit their interest to their own model, but rather they have integrated a comparative approach by analysing phylogenetic relationships among 92 described L. plantarum strains.

Overall, the study is novel and delivers important discoveries of a longitudinal, well-replicated experiment, generating a substantial amount of genomic data. It highlights an important dimension of research that environmental selection operates at the subspecies level.

Weaknesses:

Even though the authors show only one particular example by conducting their longitudinal experiment, they honestly acknowledge failures important for interpretation of the biological significance of the results (gnotobiotic mono-association experiments was done with *D. melanogaster*, but not *D. simulans*) and therefore they state limitations of their conclusions (weaker effects in the non-axenic flies are due to the presence of other taxa or to higher-order interactions with other members of the microbiome). These interactions could significantly affect bacterial growth, metabolism, and physiological influence on the host.

The authors exploit the results of their experiment to speculate about a wide range of evolutionary phenomena, like within-species competition, ecological adaptation and evolution of the host, fitness advantage of bacteria to the host, the benefits of parasitism or mutualism, the domestication of the microbiome, etc. At the end, they conclude that their study "highlights that even subspecies diversity plays a key role in adaptation to environmental temperature". However, the potential mechanisms of such adaptation are barely discussed, so that the focus of the study shifts from the temperature-induced changes in microbial population structures toward metabolism-related adaptations of clade representatives that enable them to diversify their carbon and nitrogen sources. The role of the temperature factor remains elusive.

In addition to that, the paper has a clearly minimalistic experimental approach to address functional properties of the revealed L. plantarum strains, so that their own fitness, or their relationship with the Drosophila host, is characterised superficially. Therefore, the authors' discourse can be speculative rather than factual (especially when the authors use the expression "likely" to share their guesses in the "Results" section). Nevertheless, these minor drawbacks do not underscore the novelty of the discovered phenotypes and the importance of their further investigation.

Comments on revised version:

I have read the authors revisions and find them compelling and they address fully the minor points raised in my review.

# Author comment: Author response

**DOI:** 10.7554/eLife.110808.3.sa4

**Authors:** Juan Bosco Gracia Alvira, Stefanie Migotti, Xiaomeng Tian, Viola Nolte, Christian Schlötterer

**Affiliations:** University of Veterinary Medicine Vienna Vienna Austria; University of Veterinary Medicine Vienna Vienna Austria; University of Veterinary Medicine Vienna Vienna Austria; Vetmeduni Vienna Wien Austria; University of Veterinary Medicine Vienna Vienna Austria

The following is the authors’ response to the original reviews.

**Public Reviews:**

**Reviewer #1 (Public review):**

Summary:

The factors that create and maintain diversity in host-associated microbiomes remain poorly understood. A better understanding of these factors will help in the efforts to leverage the adaptive potential of the microbiome to help solve pressing problems in health and agriculture.

Experimental evolution provides a promising path forward as we can track the causes and consequences in the emergence of novel variants, but experimental evolution remains underutilized in host-microbiome interactions. Here, Gracia-Alvira utilizes a long-term experimental evolution study in *Drosophila simulans* under hot and cold temperature regimes to identify strain-level variation in an important fly bacterium, *Lactiplantibacillus plantarum*. They identify three strains of *L. plantarum*, which are most prevalent in their respective three temperature regimes, suggesting that these are locally adapted bacteria. Then, using a combination of genomics, in vitro, and in vivo, Gracia-Alvira et al attempt to understand the factors that led to the differentiation of the hot and cold *L. plantarum* and their impacts on the fly host.

Strengths:

This is an excellent use of experimental evolution to track the emergence of novelty in the microbiome. The genomic analyses are all solid and appropriate for the data sets. It is especially striking that the comparisons with the other, independent experimental evolution studies in different labs (and across continents between Portugal and South Africa) show a consistent response to temperature. Many have disregarded the microbiome as it is something that is too sensitive to seemingly innocuous variables (particularly in the fly microbiome), such that we cannot find generalities. However, this finding highlights the potential for experimental evolution to uncover these dynamics. The question of how strains emerge and are maintained is timely and is one of the key open questions in host-microbiome evolution currently.

Weaknesses:

(1) The framing in the title and throughout the discussion about "subspecies competition" does not match the data that was collected. The subspecies competition requires actually tracking the competitive outcomes between the hot, cold, and unevolved *L. plantarum*. In the in vivo work, I can see that mixes of the strains were made, but they did not track whether the cold strain outcompeted the hot strain in vivo under cold conditions, for example.

We thank the reviewer for the honest concern and take this opportunity to defend our claim of "subspecies competition used across the manuscript. As the reviewer states, subspecies competition requires tracking the competitive outcomes between the three clades, and this is what we did by sampling and sequencing across ten years of experimental evolution (Figures 4 and S3). For this reason, we point that the subspecies competition assessment comes from the direct observation of changes in relative abundance across the time series, and not from the follow-up experiments in vivo or in vitro.

While Figure 4 is suggestive that there is ongoing competition in the hot temperature regime, this is not necessarily shown in the cold, which is dominated by the C clade. It could also be that the bacteria cannot survive in the flies at the different temperatures. The growth curve assays hint that the bacteria can grow, but the plate reader couldn't actually maintain the 18 {degree sign}C temperature (line 455). So all of this evidence is very indirect and insufficient to say that strain competition is driving these patterns.

We thank the reviewer for the alternative hypothesis that could explain the observed subspecies dynamic. We rule out that dominance of clade C in the cold occurs because the other two clades cannot grow in this regime based on three pieces of evidence:

(1) In the time series, clades H and U decrease, but never disappear (Figures 4 and S3), even showing some peaks of abundance in specific replicate populations (Figure S3).

(2) We isolated individuals belonging to clade H in the cold-evolved populations, as shown in figure 2. This is a direct evidence that clade H prevails in the cold-evolved populations, although in low abundance.

(3) We did grow the three taxa in fly food Petri dishes incubated at both temperature regimes, observing growth in all cases.

We will include the food growth experiment in the revised manuscript as further supporting evidence for growth in both regimes.

(2) The in vivo results are interesting in that there appears to be a fitness cost of clade C, but the explanation is underdeveloped. I say under-developed because in Figure 4, the cold *L. plantarum* remains much higher throughout adaptation to the hot temperature regime than the hot *L. plantarum* in the cold regime. The hot *L. plantarum* is low abundance throughout the cold regime. I felt like this observation was not explained, but it seems relevant to understanding the strain dynamics.

We acknowledge that a strong fitness cost of clade C is observed in axenic *D. melanogaster*. In the native host, *D. simulans*, with reduced microbiome, we observed delayed development that could even be an advantage depending on the situation, as pointed out by reviewer 3 in the recommendations.

Even if we assume that flies colonized with clade C are less fit in the experimental evolution, another caveat is whether the flies can actively select for the *L. plantarum* clade. Under this assumption, a clade that imposes a fitness cost to the fly (clade C) should be selected against over time because the flies colonized by this clade will have less offspring or develop later than the rest. Alternatively, as the microbiome is shared among all the individuals in the population, the host might not be able to “purge” the pernicious clade, and *L. plantarum* dynamics might be controlled solely by the relative fitness between clades in the given experimental treatment. We will discuss this hypothesis in the revision as a way to explain the relationship between the abundance of each clade and the effect on the host.

I will also note that this is not the first time that *L. plantarum* or other Lactobacillus have been shown to exert fitness costs to *Drosophila*. Gould, PNAS, 2018, shows that both *Lactobacillus plantarum* and *Lactobacillus brevis* in mono-association have lower fitness (measured through Leslie matrix projections using lifespan and fecundity) than axenic flies. Many studies of wild *Drosophila* fail to find Lactobacillus, or it is low abundance (e.g., Chandler, PLoS Genetics, 2014; Wang, Environmental Microbiology Reports, 2018; Henry & Ayroles, Molecular Ecology, 2022; Gale, AEM, 2025). This might help provide useful context for the in vivo results.

We thank the reviewer for the references. These observations are compared to our phenotypic results and discussed in the revised version of the manuscript.

(3) The data in Figure 4 are compelling to focus on the *L. plantarum* variants. However, I can see from the methods that the competitive mapping included only other strains of *Wolbachia*.

We appreciate the thorough reading of the methods by the reviewer. The competitive mapping comprised two steps: first we discarded the reads that mapped to *Drosophila*, *Wolbachia* and additional potential contaminants from sequencing facitilies (human, dog...). This step leaves the reads originated from whole the external microbiome of the flies, including *L. plantarum*. The second competitive mapping step recruits the reads that map any clade of *L. plantarum*.

It is not clear how other members of the microbiome changed in response to the temperature regimes. As I note in point #2, given that Lactobacillus is often rare, it is not clear what the rest of the microbiome looks like over the course of adaptation. Indeed, it seems like Mazzucco & Schlotterer, PRSB, 2021 did a broader analysis of the microbiome and found that Acetobacter is by far the most common bacterium (I think this data is also part of the data shown here?). Expanding on why or why not in this context is important and will improve this study, particularly if the focus is on connecting these evolutionary dynamics to ecological competition to explain the emergence of strain diversity.

We acknowledge that the rest of the *Drosophila* microbiome is not addressed in this study, as we wanted to focus the storyline around the intraspecific dynamics found in *L. plantarum*. We consider that a complete characterization of the whole *Drosophila* microbiome would unnecessarily elongate the paper and thus we treat it as a constant biotic factor.

We must point out that our dataset is not the one reported by Mazzucco & Schlötterer, which was done in *D. melanogaster*, rather than *D. simulans*. Nevertheless, both experiments share the same infrastructure, temperature regimes and fly maintenance.

We have included a list of taxa that were isolated from the populations, as well as to report *L. plantarum* prevalence and abundance across the experiment in order to provide context of the microbiome, beyond *L. plantarum*, to the readership.

**Reviewer #2 (Public review):**

Summary:

In this manuscript, Gracia-Alvira et al. investigated how environmental temperature affects competition among members of the microbiome, with a focus on intraspecific diversity, using the *Drosophila* model.

Notably, the authors identified three clades of *Lactiplantibacillus plantarum* from a natural population of *Drosophila simulans* collected in Florida. They tracked the dynamics of these three bacterial clades under two temperature conditions over the course of more than ten years. Using comparative genomics and phylogeny, they showed that these three bacterial clades likely adapted to their host independently in a temperature-specific manner. Further, by combining in vitro culture and in vivo mono-association assays, they demonstrated the functional divergence of these three bacterial clades phenotypically, including their growth dynamics and effects on host fitness. Lastly, they performed pathway analysis and speculated on key genomic variance supporting such functional divergence.

Strengths:

The laboratory evolutionary experiment in response to cold or hot environmental temperature is impressive, given its more than ten years of experimental time period. This collection of achieved microbiome samples paired with the fly host data can be a valuable resource for the field.

Weaknesses:

The laboratory evolutionary experiment can be limited due to its artificial experimental setup. For example, wild flies rely on a more diverse set of food sources and are constantly exposed to new bacterial inoculations, whereas under laboratory conditions, flies live in a more restricted ecosystem. In addition, environmental temperatures differ among different locations, but they also involve seasonal changes within the same region. This manuscript can be strengthened with further discussions that elaborate on these limitations.

As the reviewer has correctly noted, our experimental setting is not exempt from limitations. Lab-reared flies are fed with a defined standard diet. Furthermore, although the system is not completely closed to bacterial migration, this is limited as replicate populations are not allowed to mix during the maintenance of the flies. For this reason, we consider our laboratory setting as a compromise between observing wild populations, which undergo all biotic and abiotic stresses but cannot be manipulated, and evolving the bacteria in absence of the host, or in gnobiotic hosts, in which biotic interactions are not fully considered. We will extend on this in the new version of the manuscript.

Moreover, the extent of host effects involved in these experiments remains ambiguous, because it is unclear whether these *Lactiplantibacillus plantarum* mostly reside within fly guts or on *Drosophila* medium. The laboratory evolutionary experiment possibly favored better colonizers on *Drosophila* medium under either cold or hot temperatures, which subsequently can saturate fly guts. As fully dissociating these variables can be experimentally tedious, the authors may want to comment more on these aspects in the discussion. Or they may want to consider some measurements. For example, measuring the growth rate of these bacteria on *Drosophila* medium under different temperatures, in addition to the current MRS culture experiments, or measuring the portion of the Lactiplantibacillus on *Drosophila* medium versus these stably colonizing fly guts.

The reviewer's point was briefly addressed in the Results chapter: "Phenotypic differences in liquid culture".

**Reviewer #3 (Public review):**

Summary:

The study presents an analysis of 297 pangenomes derived from 20 populations of *Drosophila* simulans, at 19 time points for fast-reproducing individuals in a hot environment, or at 10 time points for slow-reproducing individuals in a cold environment, over a period of more than 10 years. The authors select a particular microbial component of the pangenomes and study the dynamics of *Lactiplantibacillus plantarum* strains in two environments. They discover that the revealed operational taxonomic units could be divided into three phylogenetic clades, which have their own genomic and genetic features, different adaptive capabilities that depend on the environment, and have a distinct impact on the fitness of the host.

Strengths:

The authors prove that bacterial microbiome components are sensitive to the environment and could rapidly (years) be fixed in eukaryotic populations. This study establishes a tractable model that potentially enables the study of variability of the physiological influence of distinct strains of an important commensal species, *Lactiplantibacillus plantarum*, on the *Drosophila* host. It is clearly shown that this single species consists of several phylogenetically and functionally diverse strains. The authors did not limit their interest to their own model, but rather they have integrated a comparative approach by analysing phylogenetic relationships among 92 described *L. plantarum* strains.

Overall, the study is novel and delivers important discoveries of a longitudinal, well replicated experiment, generating a substantial amount of genomic data. It highlights an important dimension of research that environmental selection operates at the subspecies level.

Weaknesses:

Even though the authors show only one particular example by conducting their longitudinal experiment, they honestly acknowledge failures important for interpretation of the biological significance of the results (gnotobiotic mono-association experiments was done with *D. melanogaster*, but not *D. simulans*) and therefore they state limitations of their conclusions (weaker effects in the non-axenic flies are due to the presence of other taxa or to higher-order interactions with other members of the microbiome). These interactions could significantly affect bacterial growth, metabolism, and physiological influence on the host.

We agree with the reviewer in that the use gnobiotic animals is a limitation, as by "tuning" the flies' microbiome we are modifying the interactions between members, which can potentially change the phenotypic outcome. Nevertheless, we use it as a complementary approach, rather than the only inference in our study.

The authors exploit the results of their experiment to speculate about a wide range of evolutionary phenomena, like within-species competition, ecological adaptation and evolution of the host, fitness advantage of bacteria to the host, the benefits of parasitism or mutualism, the domestication of the microbiome, etc. At the end, they conclude that their study "highlights that even subspecies diversity plays a key role in adaptation to environmental temperature". However, the potential mechanisms of such adaptation are barely discussed, so that the focus of the study shifts from the temperature-induced changes in microbial population structures toward metabolism-related adaptations of clade representatives that enable them to diversify their carbon and nitrogen sources. The role of the temperature factor remains elusive.

We acknowledge that our study does not fully resolve the mechanism by which a different clade ends up dominating each temperature regime. The MRS liquid experiment was an attempt to answer whether differences in optimal growth temperature could explain the temperature-specific abundance of the two clades. Our experiments showed, however, that this was not the case. Beyond this point, it is hard to disentangle the role of the temperature, as it could also act indirectly on the bacteria, for example, through the host or the food.

A second observation in our time series was that a third clade, U, was unfit in both regimes despite starting the experiment in high abundance. For this reason we also studied what made this clade less fit. Based on our analyses, we propose that the decrease of clade U was driven by the shift to a laboratory diet, shared by all experimental populations.

In addition to that, the paper has a clearly minimalistic experimental approach to address functional properties of the revealed *L. plantarum* strains, so that their own fitness, or their relationship with the *Drosophila* host, is characterised superficially. Therefore, the authors' discourse can be speculative rather than factual (especially when the authors use the expression "likely" to share their guesses in the "Results" section). Nevertheless, these minor drawbacks do not underscore the novelty of the discovered phenotypes and the importance of their further investigation.

We consider the reviewer's concern and toned down the phrasing when reporting our findings in the revised version of the manuscript.

**Recommendations for the authors:**

**Reviewer #1 (Recommendations for the authors):**

(1) One solution to resolve the "competition" issue would be to check that the *L. plantarum* strains are established at similar or different titers in the in vivo work. Fly phenotypes can be sensitive to microbial load (Keebaugh, iScience, 2018), which might explain some of the counterintuitive in vivo results. In line 227, the authors mention that "bacterial load" is contributing to the magnitude of the effect, but I don't see the data reported anywhere. If this is from the in vitro assays, then the authors need to show that in vitro predicts in vivo *L. plantarum* abundance.

Bacterial load inoculated in the in vivo experiment was normalized to OD=0.05 (~5*10^6^ CFUs/ml) for the three clades at the beginning of the experiment. Thus, all vials were inoculated with the same titer of *L. plantarum*. Only the genotype varied between treatments. However, it is possible that, once inoculated, each clade grew to a different titer (as they have different growth rate and carrying capacity).

Statement in line 227 comes from the differing results in transfers 1 and 2. In transfer 1 we inoculated a fixed load of ~2.5*10^5^ CFUs. In transfer 2, however, we inoculated no bacteria to the food, and the flies seeded the vial. Our statement comes from the assumption that bacterial load in transfer 2 has to be lower than in transfer 1 as bacteria seed the vial solely by defecation of the parents.

Following the reviewer's suggestion, in the revised version of the manuscript we have included a new experiment in which we quantified the bacterial load of each clade in individual flies.

(2) Tracking the competitive outcomes is tricky, though it could be done with whole genome sequencing. An alternative would be to label the strains with fluorescent proteins (e.g., Obadia Current Biology 2018 has done this in Lactobacillus) and track fluorescence to better understand the results of the "mix" treatment in Figure 6.

We appreciate the feedback of the reviewer, but consider this rather labour-intensive approach as an interesting option for future follow-up experiments.

(3) That being said, my main concern with this is the "competition" claim. If the paper were reframed appropriately, this paper could still make an important contribution to the evolution of host-microbiome interactions, but the authors would need to consider what they can and cannot do with this interesting dataset.

The "competition" claim comes from the changes in relative abundance observed in the time-series data, not from any of the follow-up experiments. Thus, we consider the use of the term "competition" appropriate.

(4) The text on the figures is very small and hard to read.

We increased the size of the text in all figures.

**Reviewer #2 (Recommendations for the authors):**

(1) Have you conducted the in vitro culture experiments following the "cold" conditions?

We have conducted the experiment in "cold" conditions, but with some modifications to the experimental settings, as the plate reader did not have cooling capacity. Instead, we grew a subset of the isolates (four per clade) in glass vials at constant 20 °C, and measured their OD twice a day. We have included the results in the revised version of the manuscript.

(2) How many technical and biological replicates were measured for the in vitro culture experiments (Figure 5)? Please add this information to the figure legend and method.

We measured the growth of four isolates from clade U, nine isolates from clade C and sixteen isolates from clade H. Each isolate was grown three times.

We have included this information, as requested by the reviewer.

(3) Making the labels in Figures 2, 3, 5, and 6 bigger would be helpful.

We have increased the font size of all figures.

**Reviewer #3 (Recommendations for the authors):**

(1) Line 268: "Based on our results in experimentally evolved fruit flies, we propose that within-species competition, thus far largely overlooked, could contribute to ecological adaptation and evolution of the host". Overstatement should be avoided, since the evolution of the host was not directly studied here.

Our results show that reproductive traits of the host differ upon colonization with each clade. Although we don't test the host's evolution, we speculate that flies differing in their offspring number and developmental time might differ in their overall fitness. Finally, we consider the Discussion section as the right place for speculation and development of hypotheses that can be tested in future work.

(2) Line 258: "These differences do not explain the clade-specific selection, but reflect the different evolutionary histories of the clades". The temperature factor and its possible role in clade selection would be better discussed at least a little bit.

In this paragraph we described potential metabolic differences between clades using comparative genomics. We did not find enrichment in a function or group of functions that could explain the different dynamics between clades H and C in the temperature regime.

In the revised version of the manuscript we highlight that we did not find temperature-specific differences from this analysis.

(3) Line 252: "...This could explain why clade U, which displayed a high growth rate and carrying capacity in liquid culture". The statement could be further developed with a caution. Even if the isolates that belong to the clade U are outcompeted by H or C, it should be noted that the strain U cannot be used as a true reference for fitness, since it could possess its hidden adaptive properties, not being simply "a loser". Such a hypothesis could explain the maintenance of this strain in the wild.

We agree with the reviewer in that fitness is relative to the selective environment. Clade U is less fit than H and C in our specific experimental conditions, but it could outcompete them in other conditions, such as wild flies or MRS liquid medium. In the revised version of the manuscript we have rephrased this statement to clarify that we specifically refer to clade U's fitness under the new laboratory conditions.

(4) In a cold environment, association with the clade C induces developmental delay and produces less progeny, which potentially allows the host to survive in case of harsh conditions and potential food limitation. Could the authors speculate and not exclude that this phenotype could be potentially adaptive? It would be curious to check in further studies whether flies associated with C strains are more stress-resistant, for example.

We thank the reviewer for this alternative hypothesis. In our manuscript we used the Darwinian definition of fitness; reproductive success of an organism in the focal environment. And thus, both higher progeny per female and shorter developmental time would be beneficial in direct competition with other individuals. It is true that delayed developmental time, or less progeny, could be advantageous in specific cases. This could be the case for *D. simulans* inoculated with clade C. However, we consider that the fecundity levels observed in *D. melanogaster* upon inoculation with Clade C (average of 0.06 offspring/female*day in the cold) are too low to sustain a population.

We have included this hypothesis in the Results section.

(5) It would be highly recommended to add an experiment to complete the story by measuring the quantity of bacteria in the medium and in the flies. This will resolve the hypothesis (Line 785): "Thus, the ability to exploit this ubiquitous source of carbon and nitrogen could be very advantageous in the fly microbiome context, but would not affect the fitness in liquid culture".

Following the reviewer's recommendation we included two additional experiments. We measured the bacterial load per fly in the native host, *D. simulans*, inoculated with the three clades. We also compared the clades' growth speed in solid fly food (without host). In the former experiment, we found similar bacterial loads upon inoculation with clades U and clade H. In contrast, in the latter we found delayed growth of clade U relative to H and C in the food. Thus, chitobiose consumption does not seem to provide an advantage in the fly gut to clade H. We attribute the fitness advantage of H and C to their advantage growing on the laboratory fly food, regardless of the host.

Both experimental results have been included in the revised version of the manuscript, and the comparative genomics paragraph and discussion have been modified in consequence.

(6) The chapter "Extended clade-specific differences in KEGG metabolic pathways" could be presented in the main text as it contains important results. These results are mentioned in the chapter "Functional divergence on the genomic level", which looks rather humble when it stands alone as it currently does.

We appreciate the interest of the reviewer in this supplementary chapter. To keep the length of the manuscript digestible for a broad set of readers, we decided to only include in the main text the functional differences that could play a role in adaptation to the new laboratory environment.

We consider that a full description of the metabolic differences between the three clades has to be published, as it might be relevant for researchers interested in *L. plantarum* metabolism. However, it does not fully follow the storyline, as the differences reported in the supplementary, such as nitrate respiration or synthesis of molybdenum cofactors, might not be involved in the clade-specific selection observed in the time series.

(7) Line 773: "Clades C and H encode a shared genetic repertoire related to sugar/riboflavin metabolism that is lacking in clade U". This indeed allows us to hypothesise that the fixation of these clades in fly populations was due to their improved metabolic capabilities. However, the analysis of fitness shows similarity in flies associated with clades H and U, meaning that sugar/riboflavin metabolism in H does not provide an obvious adaptive trait to flies. Moreover, one could say that sugar metabolism in clade C is maladaptive not only for flies, but also for bacteria in liquid cultures. It is recommended to more clearly state the respective limitations of the study.

Here we have to make a distinction between bacterial fitness and host fitness. The three clades differ in their (bacterial) relative fitness, as evidenced by the time-series dynamics (Figure 4). In the cited statement we hypothesize that a more versatile sugar metabolism repertoire could increase the bacterial fitness of clades H and C (relative to clade U) in the sugar-rich laboratory diet.

This is independent of the fitness effect that *L. plantarum* could have in the host. Finally, as it was discussed in the recommendation 3, fitness is specific to the environment. Clade C is the least fit in liquid MRS in hot conditions, but the fittest in cold experimental conditions.

(8) The authors should better explain why growth in MRS was not performed in a cold temperature regime to further support or refute the hypothesis that capacity and inflection time could partially explain the higher fitness of bacterial strains from clade U.

We did not perform this experiment in cold conditions due to technical limitations of the plate reader, that does not have cooling capacity. Nevertheless, following the reviewers' suggestion, we have included in the revised version of the manuscript a new MRS growth experiment in cold-like conditions (constant 20 °C).

(9) When mentioning that *L. plantarum* can "increase larval fitness of *Drosophila melanogaster* relative to germ-free flies" (line 196), the authors should specify in which specific conditions this phenotype was observed, and how relevant the mentioned phenotypes are to the current study.

Following the reviewer's recommendation, we have modified the paragraph in order to clarify the conditions used in other papers and those used in our work. The references cited in this section (PMID: 21907145, 29290388 and 28062579) report that *L. plantarum* increases the host fitness in protein-poor diets (12 g/l of dried yeast or less), but not in high-protein diet (50 g/l of yeast or higher). Since our experimental diet contains an intermediate amount of protein (24.3 g/l of dried yeast) we were agnostic of whether *L. plantarum* would benefit the host or not in our conditions. Regarding the phenotypes, we chose two reproductive traits that are affected by changes in the microbiome according to the literature. Developmental time is directly affected by *L. plantarum* in the aforementioned papers. Offspring number is another fitness component affected by *Drosophila* microbiome (PMID: 30510004).

(10) Provide a reference for line 205: "In axenic *D. melanogaster* none of the *L. plantarum* clades provided a fitness advantage to the host relative to germ-free controls, contrary to the effects reported in the literature". If the conditions were different from those in the studies referred to, then it would be of no use to compare the fitness advantage (for example, in Reference 24 another type of diet was used).

Already covered in recommendation 9.

(11) Please provide more context to this statement (Line 210): "The high content of dried yeast 24.3 g/l in the fly food used in our experiment likely provided already sufficient amounts of essential amino acids, which negated the growth-promoting effects of *L. plantarum*". It is not clear why amino acids are taken into account, and what the evidence is for the fact that the amount of essential amino acids was sufficient to abolish growth-promoting effects.

The whole paragraph was modified in order to clarify the relationship between protein input and nutritional fitness benefit of *L. plantarum*.

(12) Please provide measurements of bacterial quantity which would support the statement (Line 215): "The fitness reduction was stronger in the first transfer of flies, likely due to a higher bacterial load".

Upon request of the reviewer, we have estimated the bacterial load per individual fly in *D. simulans*. Additionally, we have specified the CFUs inoculated in the vials in transfer 1.

(13) Correct the typo (line 220): "However, the developmental time was significantly extended after inoculation with clade C at cold temperature (Dunn's test, p < 0.05 05 for all significant comparisons)".

Done.

(14) Specify more precisely the temperature conditions referred to in line 226: "In summary, we observed that clade C, which is dominant in the cold-evolved populations, decreases host fitness when axenic flies are inoculated". Does it decrease fitness both in hot and cold environments?

For the axenic flies, we did find a decrease in fitness in both regimes, yes. We specified it in the revised version of the manuscript.

(15) Please provide evidence for line 227, or otherwise rephrase it: "The magnitude of this effect varies depending on the environmental temperature, the bacterial load, and the presence of other microbial taxa".

Novel evidence was provided regarding the role of bacterial load on host fitness.

(16) Correct the following statement, so that it reproduces the results of the original work (reference 19, line 229): "In a low-protein diet, strains that were not isolated from *Drosophila* enhanced larval growth relative to germ-free individuals, whereas another *Drosophila*-associated strain did not have any effect".

This statement was removed from the revised version. This reference was cited in the discussion to state that: " the nutritional symbiosis in *L. plantarum* is strain-specific".

(17) Please provide a rationale for using KEGG Orthologs. Why was this database chosen as an appropriate one, even though it is known to be a non-exhaustive metabolomic resource?

KEGG is a well-known metabolic database that is widely used in comparative genomics (PMID: 40177264) and built in state-of-the-art software for microbial ecology such as Anvi'o (PMID: 33349678). Other similar gene-to-function databases are less focused on metabolic pathways, such as COG or GO, or limited to specific enzymatic activities, like CAZy. Furthermore, the hierarchical organization of KEGG Orthologs in modules and pathways allowed us to map clade-specific orthologs to the broad metabolic context. For these reasons, we considered KEGG to be the best option for this analysis.

(18) Line 250: "Therefore, we speculate that the ability to exploit this ubiquitous source of carbon and nitrogen in the lab-maintained fruit flies, could be a strong target of selection in the lab environment". This statement concludes the "Results" section but would be more appropriate for the Discussion section, since the authors do not provide any experimental evidence that could support this statement.

We have modified this chapter, as covered in recommendation 5.

(19) Line 276: "However, the intraspecific richness of L.plantarum in our flies was three times higher than that estimated in human gut microbiomes". Note that there are other recent studies which show the presence of several OTUs within L.plantarum isolates (for example PMID: 41484402).

We thank the reviewer for the reference. We comment on it in the revised manuscript.

(20) Line 287: "Our finding shows that the well-characterized nutritional symbiosis between *Drosophila* and *L. plantarum* depends on the bacterial genotype and cannot be generalized to the entire species". Note that such a conclusion has already been previously stated (for example, PMID: 30008290 and 28993620).

We thank the reviewer for the references. Indeed, these papers show that some *L. plantarum* strains are beneficial for the host while others are neutral. Furthermore, as commented by Reviewer #1 in the public review, *L. plantarum* has been shown to reduce the host's fitness relative to axenic flies (Gould, PNAS, 2018).

Our observations are novel in two ways. (1) The fecundity observed in *D. melanogaster*, 0.06 offspring/female/day in average, is lethal (in Gould et al. 2018 fecundity never decreased below 1 offspring/female/day). (2) Clade C outcompetes the other clades in the cold, despite being detrimental for the host.

We have modified the Discussion to account for the previous work.

(21) Line 343: "In addition, we obtained *L. plantarum* genomes from two other experimental evolution studies. Two genomes from the South African experiment and seven genomes from the Portugal experiment". Merge two sentences into one.

Done.

(22) Line 360: "At sampling, the age of the flies varied between four and eight days for the hot environment and between nine and 16 days for the cold environment". Please comment on the fact that different age of flies (different physiology) is not the reason for bacterial community differences.

During maintenance, flies are sampled at different ages because the temperature affects their developmental time. We cannot rule out the hypothesis that age difference drives microbiome differences. Temperature could affect clade competition directly (differences in optimal temperature between clades) or indirectly, by affecting either the host (e.g. changes in *Drosophila* developmental time alters *L. plantarum* fitness), the surounding microbiome, or the food (e.g. increased metabolic activity in the hot regime changes nutrients profile). We ruled out the direct effect of temperature with growth experiments in liquid MRS medium and solid fly food, but disentangling the indirect effects is not feasible.

(23) The majority of figures have low-quality labels that are not legible due to the small size of the font. Please improve.

Done.

(24) Figure 1 - Correct the legend: There is no "10" label on the picture. Probably by 10, the authors mean "Generation", while by x10 - number of isogenic replicates.

Done.

(25) Figure 2 - No numbers at nodes are indicated, whereas it is announced in the legend that they represent bootstrap support values. In addition, it is recommended to show a reference pangenome in the middle panel to clearly refer to the total size of the possible black bar.

We added high bootstrap support as coloured nodes in figures 2, 3 and S2.

We do not understand the reference pangenome request. In the middle panel, each black/white bar corresponds to an orthologous gene that can be either present or absent in each of the genomes. These orthologs were sorted based on hierarchical clustering of the their patterns of abundance (columns present in the same set of genomes, together), not by synteny. Thus, a reference pangenome would be simply a black bar.

(26) Figure 2: It would be advantageous to add a figure that represents the frequency of each strain in each replicate (at the last time point, for instance). It would explain why some “blue” strains appear to be within the “red” cluster. Otherwise, it is confusing to find cold-evolved bacterial strains in hot-evolved fly populations.

The frequency of each clade in each replicate is shown in figure 4. We think that it would be more confusing to follow the suggestion of the reviewer, as the isolates were sampled at different time points of the experiment. We would not like to call it a confusion that "blue" strains appear in the "red" cluster, but rather the logical consequence of the color code used in figure 2, which corresponds to the temperature regime in which the isolate was sampled (regardless of its clade). Whereas in the following figures colour represents the clade. It is thus possible to find clade H isolates in the cold temperature regime, as this clade is in low frequency but not completely absent in this regime.

(27) Figure 3 – Add a label for the X-axis.

We rotated the tree to be able to increase the genome IDs. We have added the label to the Y-axis.

(28) Figure 4 - Please indicate how the clade relative abundance was assessed.

Clade relative abundance was inferred by mapping competitively the short reads against the three clades’ reference sequences. It is specified in the legend now.

(29) Figure 6 - Total number of F1 flies eclosed normalised by day (during which period?). What do T1 and T2 correspond to?

During the respective number of days that females were allowed to lay eggs: one day in the hot settings and two days in the cold settings in transfer 1. One day and three days, respectively, in transfer two.

T1 and T2 correspond to the first and second transfers, as described in the Materials and Methods. In first transfer, flies laid eggs in vials pre-inoculated with a set load of *L. plantarum*. After egg laying, same adults were then transferred to a sterile set of vials and allowed to lay eggs again (second transfer). Bacterial load in these vials was solely seeded by the parents.

In order to avoid any confusion, in the revised version of the manuscript we have modified figure 6 to show transfer 1 for both *Drosophila* species, and moved transfer 2 dataset to supplementary figure S5.

(30) Figure S2 - label the X-axis.

We guess the reviewer means Y-axis. Done.

(31) Figure S3 demonstrates the real data and its variability, so it would be better used instead of Figure 4 (which seems to be just a derivative from Figure S3, not a separate dataset and separate type of analysis).

As the reviewer suggested, we have replaced figure 4 with figure S3.

(32) Figure S4: Improve plot title: (e.g., C:H:U = 3:3:3).

Done.

(33) Figure S6: It is stated that N = 10; however, some datasets do not have 10 points represented. Please specify why. Also, please specify the meaning of "T1/T2".

For the inoculation experiment in *Drosophila simulans*, we had nine replicates per treatment, not ten. This has been corrected in the figure and in the Materials and Methods section.

T1 and T2 correspond to the first and second transfers, already covered in recommendation 29.

(34) Table S3: provide legend for values (1 - present in all strains, but 0.04 - what does it mean?).

It means that 4% of the genomes from this clade harbour the specific gene. We have specified it in the legend of the revised table.
